# Identification and characterization of an invasive, hyper-aerotolerant *Campylobacter jejuni* strain causing bacteremia in a pediatric leukemia patient

**DOI:** 10.1128/asmcr.00060-24

**Published:** 2025-02-26

**Authors:** Ximin Zeng, Kevin M. Lloyd, Rana F. Hamdy, Craig A. Shapiro, Mark A. Fisher, Jun Lin, Benjamin M. Liu

**Affiliations:** 1 Department of Animal Science, University of Tennessee, Knoxville, Tennessee, USA; 2 Division of Pediatric Infectious Diseases, Children’s National Hospital, Washington, DC, USA; 3 Department of Pediatrics, the George Washington University School of Medicine and Health Sciences, Washington, DC, USA; 4 Department of Pathology, University of Utah School of Medicine, Salt Lake City, Utah, USA; 5 ARUP Laboratories, Salt Lake City, Utah, USA; 6 Division of Pathology and Laboratory Medicine, Children’s National Hospital, Washington, DC, USA; 7 Department of Pathology, the George Washington University School of Medicine and Health Sciences, Washington, DC, USA; 8 Department of Microbiology, Immunology and Tropical Medicine, the George Washington University School of Medicine and Health Sciences, Washington, DC, USA; 9 Children’s National Research Institute, Washington, DC, USA; 10 The District of Columbia Center for AIDS Research, Washington, DC, USA; Vanderbilt University Medical Center, Nashville, Tennessee, USA

**Keywords:** hyper-aerotolerant, bacteremia, *Campylobacter jejuni*, invasive, pediatric

## Abstract

**Background:**

*Campylobacter jejuni* is a leading pathogen causing gastroenteritis in humans. Invasive *C. jejuni* strains crossing the gut-blood barrier can cause bacteremia. Despite its microaerophilicity (preferring <5% O_2_ atmosphere), hyper-aerotolerant (HAT) *C. jejuni* has recently been isolated from intestines in poultry and humans. Elevated aerotolerance may enhance *C. jejuni* survival in the ecosystem, thereby promoting its transmission. However, invasive HAT isolates of *C. jejuni* have not been reported in bacteremia in humans.

**Case Summary:**

A *C. jejuni* strain (CNH-HAT-1) was isolated from a 7% CO_2_ (~16% O_2_) atmosphere subculture of an aerobic blood culture from a 5-year-old boy with B-cell acute lymphoblastic leukemia presenting with bloody stool, fever, and neutropenia. The organism identification was determined by matrix-assisted laser desorption/ionization time-of-flight mass spectrometry and 16S-rRNA sequencing. HAT phenotype of this isolate was confirmed by aerotolerance testing. Belonging to the ST-21 clonal complex, CNH-HAT-1 is phylogenetically close to recently reported invasive *C. jejuni* sheep abortion strains and some bacteremia-associated strains. CNH-HAT-1 possesses a CJIE-1 prophage element, a functional enterobactin receptor CfrB, and G250A mutation in the major outer-membrane protein PorA, which likely contributes to *C. jejuni* invasiveness. CNH-HAT-1 is resistant to penicillins/cephalosporins but susceptible to aminoglycosides, fluoroquinolones, and macrolides. The patient improved with intravenous gentamicin, followed by enteral therapy with azithromycin.

**Conclusion:**

We identified a novel invasive bloodstream *C. jejuni* strain proven *in vitro* to be HAT, shedding light on key genomic features linking with its pathogenesis. Given its enhanced survival of HAT *C. jejuni* in ecosystem, a clinical microbiology lab should be vigilant with detecting them from blood cultures.

## INTRODUCTION

*Campylobacter* is a leading cause of acute gastrointestinal infection in humans in developed countries, with *Campylobacter jejuni* being the most common human pathogen within the genus (1, 2). The Centers for Disease Control and Prevention estimates there are 1.5 million people each year in the U.S. infected with *Campylobacter,* with a financial burden on the U.S. health system of $1.7 billion (3). Invasive *C. jejuni* strains crossing the gut-blood barrier can cause bacteremia, which can be life-threatening in immunocompromised patients (4). *C. jejuni* bacteremia may be under-reported, due to the absence of *Campylobacter*-specific protocols and challenges of recovering microaerophilic *C. jejuni* (preferring <5% O_2_ atmosphere) in aerobic or anaerobic blood culture bottles (5, 6).

Hyper-aerotolerant (HAT) *C. jejuni* has recently been isolated from human and poultry intestinal samples (7, 8). Aerotolerance may enhance *C. jejuni* survival in the environment, thereby increasing the risk of transmission. However, invasive HAT *C. jejuni* isolates have not been reported as a cause of bacteremia in humans to date. In this report, we identified and characterized a HAT *C. jejuni* strain, CNH-HAT-1, at Children’s National Hospital (CNH).

## CASE PRESENTATION

### Clinical case and isolation/identification of CNH-HAT-1

A 5-year-old male with B-cell acute lymphoblastic leukemia (B-ALL) on maintenance chemotherapy with mercaptopurine and methotrexate presented to the emergency department with cough and progressive abdominal pain in the setting of intermittent fevers over the preceding month. He was neutropenic at the time of admission, with an absolute neutrophil count of 160 cells/µL. He did not have exposure to poultry or sheep. He was treated empirically with cefepime for febrile neutropenia and started on azithromycin for a 5-day course of therapy for possible atypical pneumonia, given his persistent cough.

**Fig 1:**
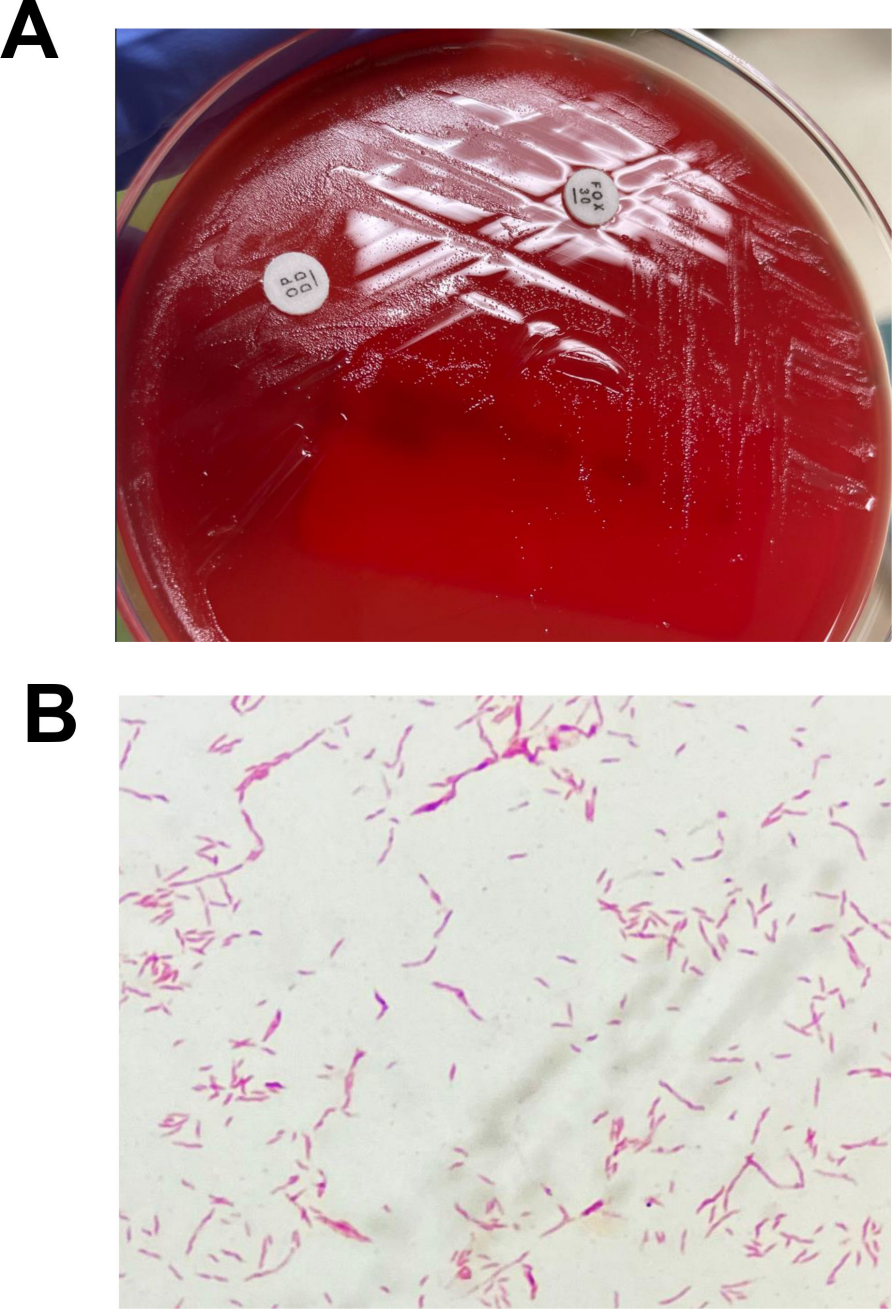
Phenotypic features of the clinical *C. jejuni* isolate CNH-HAT-1. (**A**) Gray, non-hemolytic colonies on a Columbia blood agar plate after 72 h incubation of a blind subculture of the positive blood culture broth at 35°C with 7% CO_2_. (**B**) Gram stain of the colonies from (**A**) demonstrating Gram-negative seagull wing morphology.

An aerobic blood culture was collected immediately after the admission and signaled positive after 72 h incubation in an aerobic FA Plus bottle in the BACT/ALERT VIRTUO system (bioMérieux, Durham, NC). However, Gram stain of the blood culture broth did not yield anything resembling bacteria or fungi. GenMark/cobas ePlex Blood Culture Identification Gram-Positive and Gram-Negative multiplex PCR panels (GenMark/Roche, Carlsbad, CA) yielded negative results. Blind subcultures to Columbia sheep blood and MacConkey agar were incubated at 35°C with 7% CO_2_ for 72 h until small colonies were observed (Fig. 1A). Oxidase-positive Gram-negative rods with seagull wing morphology (Fig. 1B) were identified as *C. jejuni* using MALDI-TOF MS at CNH (Bruker Biotyper, CLAIM-6-Library, 3399 MSPs) and confirmed by ARUP Laboratories using MALDI-TOF MS (Bruker Biotyper, 14600 isolate library) and 16S rRNA sequencing.

### Antimicrobial susceptibility testing (AST) of CNH-HAT-1

AST using custom Sensititre plates and Sensititre Campy 2 plates (Thermo Fisher Scientific, Kansas City, KS) showed that CNH-HAT-1 was susceptible to erythromycin, ciprofloxacin, and gentamicin but displayed elevated MICs to penicillins and cephalosporins (Table 1). The patient improved with initial empiric therapy with IV gentamicin, followed by enteral therapy with azithromycin to complete a total of 2 weeks of total therapy. He recovered well from this infection without recurrence.

**TABLE 1:** AST of the blood *C. jejuni* isolate CNH-HAT-1

Antibiotic	MIC (interpretation, if breakpoints available)
Erythromycin	0.5 (susceptible)
Azithromycin	≤0.06
Ciprofloxacin	≤0.12 (susceptible)
Levofloxacin	0.12
Tetracycline	≤1 (susceptible)
Imipenem	≤0.03
Gentamicin	≤0.25
Penicillin	≥4
Ampicillin	≥16
Amoxicillin/Clavulanic acid	2/1
Cefuroxime	≥16
Ceftriaxone	≥4
Meropenem	≤0.06

### CNH-HAT-1 is a HAT *C. jejuni* strain

**Fig 2:**
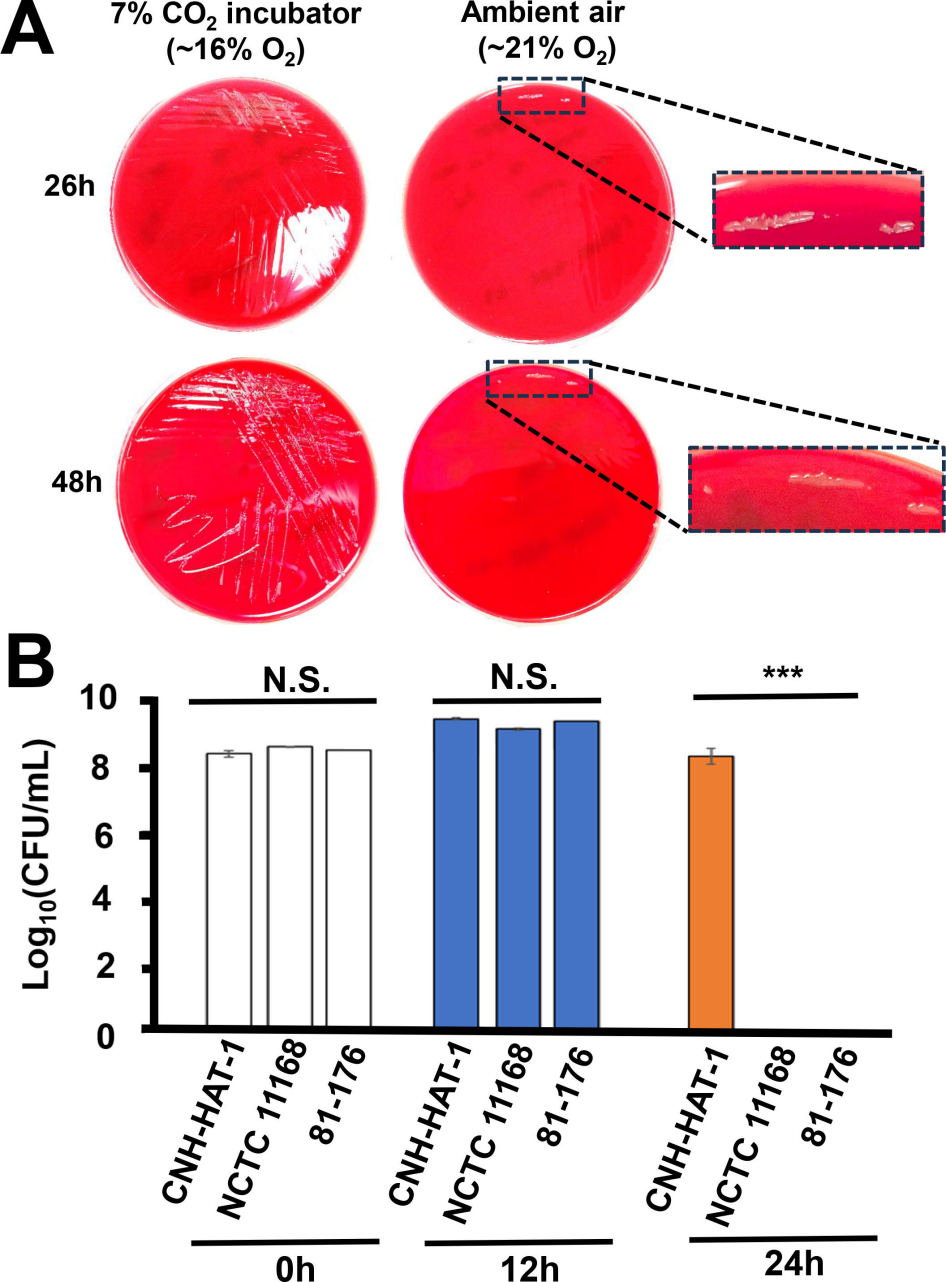
Hyper-aerotolerance phenotype of the clinical *C. jejuni* isolate CNH-HAT-1. (**A**) Colonies of CNH-HAT-1 subculture on Columbia blood agar plates after 26 and 48 h of incubation in 7% CO_2_ incubator (~16% O_2_) and ambient air (~21% O_2_), respectively. Insets of the image panels highlight the colonies on the plates with ambient air. (**B**) The clinical *C. jejuni* isolate CNH-HAT-1, isolated from an aerobic blood culture bottle, was tested for aerotolerance by comparing quantitative culture (CFU/mL) at the indicated time points after aerobic shaking at 200 rpm at 42°C, with comparison with aerotolerant *C. jejuni* control strains NCTC 11168 and 81-176. N.S., not significant; ***, *P* < 0.001.

**Fig 3:**
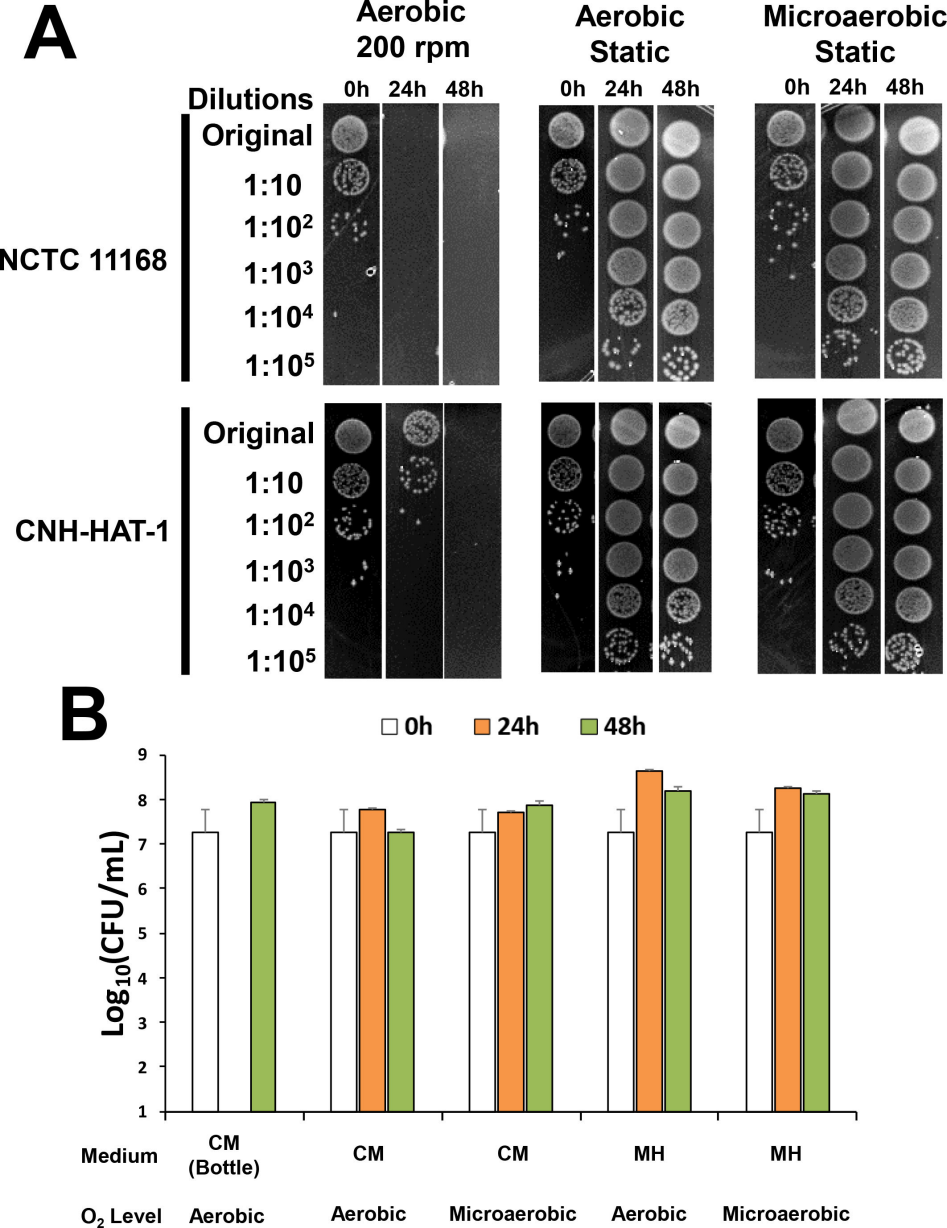
The impact of culture conditions and culture media on aerotolerance of *C. jejuni*. (**A**) Overnight fresh culture of aerotolerant control strain NCTC 11168 and hyper-aerotolerant clinical isolate CNH-HAT-1 were diluted to approximately 2 × 10^6^ CFU/mL in Mueller Hinton (MH) broth and incubated at 37°C in the indicated oxygen levels (aerobic or microaerobic) and different shaking conditions (static or shaking at 200 rpm). At the indicated different time points (24 and 48 h post-inoculations), bacteria were collected, subjected to serial dilution, and spotted on MH agar plates (2 µL per spot). The experiments were performed in duplicates. (**B**) Overnight fresh culture of CNH-HAT-1 was diluted to approximately 2 × 10^7^ CFU/mL in each test medium and incubated at 37°C under different oxygen levels. We skipped the 24 h time point for BacT/ALERT FA Plus Aerobic Culture Media (CM) (bottle) group to avoid introducing oxygen into the sealed bottle. The experiments were performed in triplicates. CM (Bottle), the BacT/ALERT FA Plus Aerobic Culture Media in an aerobic bottle (~30 mL/bottle); CM, the BacT/ALERT FA Plus Aerobic Culture Media in test tubes (5 mL/tube); MH, Mueller Hinton broth medium in test tubes (5 mL/tube).

*C. jejuni* typically grows in microaerophilic conditions only (5, 6). However, CNH-HAT-1 formed colonies not only in standard CO_2_ incubation conditions (5%–7% CO_2_, 17%–19% O_2_ at 35°C; Fig. 2A, left) but also in ambient air (~21% O_2_) (Fig. 2A, right). This prompted us to assess the aerotolerance of CNH-HAT-1 using established methods and criteria (9, 10). Aerosensitive, aerotolerant, and HAT *C. jejuni* isolates are defined based on how long they lose viability after aerobic shaking at 200 rpm (9, 10). As shown in Fig. 2B, there were no significant differences in quantitative culture (a proxy of viability) between the CNH-HAT-1 and aerotolerant *C. jejuni* control strains NCTC 11168 and 81-176 before or after 12 h aerobic shaking. In contrast, CNH-HAT-1 displayed a significantly (*P* < 0.001) higher level of viable cells after 24 h aerobic shaking than NCTC 11168 and 81-176, which completely lost its viability (Fig. 2B and 3A, left). CNH-HAT-1 was not viable at 48 h but survived at 24 h aerobic shaking (Fig. 3A, left). Therefore, CNH-HAT-1 was determined to be a HAT strain.

To compare the effects of culture media and conditions on the aerotolerance of *C. jejuni*, we performed growth experiments under static conditions. NCTC 11168 and CNH-HAT-1 both grew under aerobic and microaerobic conditions, with no significant difference (Fig. 3A, middle and right). Static cultivation permitted the survival and growth of CNH-HAT-1 at 24 and 48 h of incubation, regardless of media types (BacT/ALERT FA Plus Aerobic blood bottle culture media or Mueller Hinton broth) and oxygenation conditions (aerobic and microaerobic) (Fig. 3B).

### Whole genome sequencing (WGS) of CNH-HAT-1

Short-read (Illumina) and long-read (Nanopore) WGS of CNH-HAT-1 were performed, followed by hybrid genome assembly (11). The total assembled length is 1,786,214 bp, with 30.35% guanine-cytosine content (Table 2). Multilocus sequence typing (MLST) analysis (10) revealed the sequence type (ST) of CNH-HAT-1 is 454, which belongs to the ST-21 clonal complex (CC). This is the same CC as the invasive sheep abortion strain IA3902 (12). Interestingly, CNH-HAT-1 also possesses a G250A mutation in major outer-membrane protein PorA, which likely contributes to *C. jejuni* invasiveness and sheep abortion (13). An antibiotic resistance gene search using ResFinder 4.4.2 (14) identified the beta-lactamase resistance gene *bla*_OXA-61_ (Cj0299), with guanine to thymine transversion at 57 bp upstream of the start codon of the *bla*_OXA-61_, which leads to constitutive expression of this beta-lactamase (7). Consistent with this genetic finding, CNH-HAT-1 was resistant to penicillins and cephalosporins on phenotypic AST (Table 1). These contigs do not contain known plasmid origins of replication based on PlasmidFinder 2.1 (8).

**TABLE 2:** Sequencing statistics of CNH-HAT-1

Metric	Value
Total aligned length (bp)	1,786,214
N50 (bp)	1,307,642
N75 (bp)	306,648
L50	1
L75	2
No. of N’s per 100 kbp	0
No. of contigs	4
Largest contig (bp)	1,307,642
GC%	30.35

**Fig 4:**
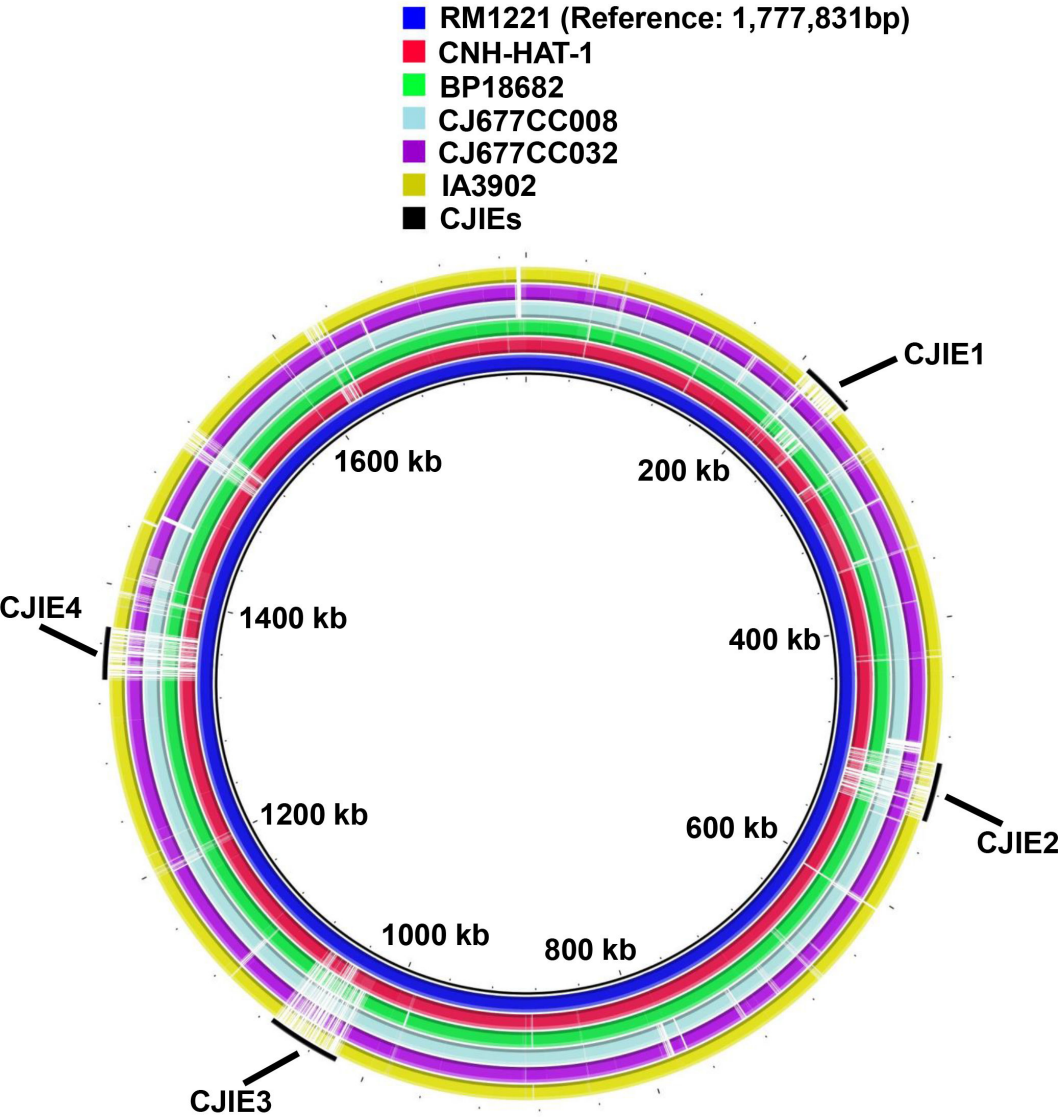
Comparative genomic analysis of CNH-HAT-1. Comparative genomic analysis of CNH-HAT-1 was performed using BRIG v0.95 and BLAST + 2.15.0, compared with the indicated reference strains. Among them, RM1221 (most inner ring, blue) is the reference strain with four *C*. *jejuni*-integrated elements (CJIE1-CJIE4) in the genome. BP18682, CJ677CC008, and CJ677CC032 are bacteremia strains. IA3902 is a representative sheep abortion strain.

Interestingly, contig_1 (38,548 bp) is syntenic with the prophage-integrated element 1 (CJIE1) chromosomal region of *C. jejuni* RM1221 (Table 3 and Fig. 4). Recently, the ST-677 CC *C. jejuni* strain, the major human bacteremia-causing *C. jejuni* strain in Finland (15, 16), was found to possess the CJIE1-like element (15). Another set of physiologically important genes of bacteremia *C. jejuni* strains is iron acquisition systems, with which bacteremia strains compete for iron from sequestering molecules/carriers in blood, such as heme and hemoglobin (17). Like ST-677 CC strains (15), CNH-HAT-1 also possesses intact ferric enterobactin receptor CfrB and a heme/hemoglobin utilization system (ChuABCD) (17) (Table 4). However, ChuABCD is universally present in different strains and therefore may not be a niche-specific determinant.

**TABLE 3:** Characteristics of contigs obtained from whole genome sequencing of CNH-HAT-1

Contig	Length (bp)	Read depth by Illumina	Syntenic region in *C. jejuni* RM1221
Contig_1	38,548	150	*C. jejuni* prophage integrated element 1 (CJIE1) of RM1221
Contig_2	171,924	138	CJE1326-1530 of RM1221
Contig_3	1,307,642	134	CJE0001-1325 of RM1221
Contig_4	306,648	134	CJE1531-1799 of RM1221

**TABLE 4:** Comparison of iron acquisition systems among CNH-HAT-1, NCTC 11168 and 81-176

Iron acquisition gene	Description	CNH-HAT-1	NCTC 11168	81-176
CfrA	Ferric enterobactin receptor	Pseudogene	+	-
CfrB	Ferric enterobactin receptor	+	Pseudogene	+
Cee	Ferric enterobactin hydrolase	+	+	-
CeuBCDE	Ferric enterobactin transporter	+	+	+
TonB1-ExbB1-ExbD1	Energy transduction system	+	+	-
TonB2-ExbB2-ExbD2	Energy transduction system	+	+	+
TonB3-ExbB3-ExbD3	Energy transduction system	+	+	No TonB3
ChuA	Heme receptor	+	+	+
ChuBCDZ	Heme transporter	+	+	+
Cj0178	Ferri-transferrin receptor	+	+	-
Cj0173c-75c	Heme transporter	+	+	+
FeoB	Ferrous iron transporter	+	+	+
Cj1658-63	Rhodotorulic acid transporter	+	+	+

### Phylogenetic analysis of CNH-HAT-1

**Fig 5:**
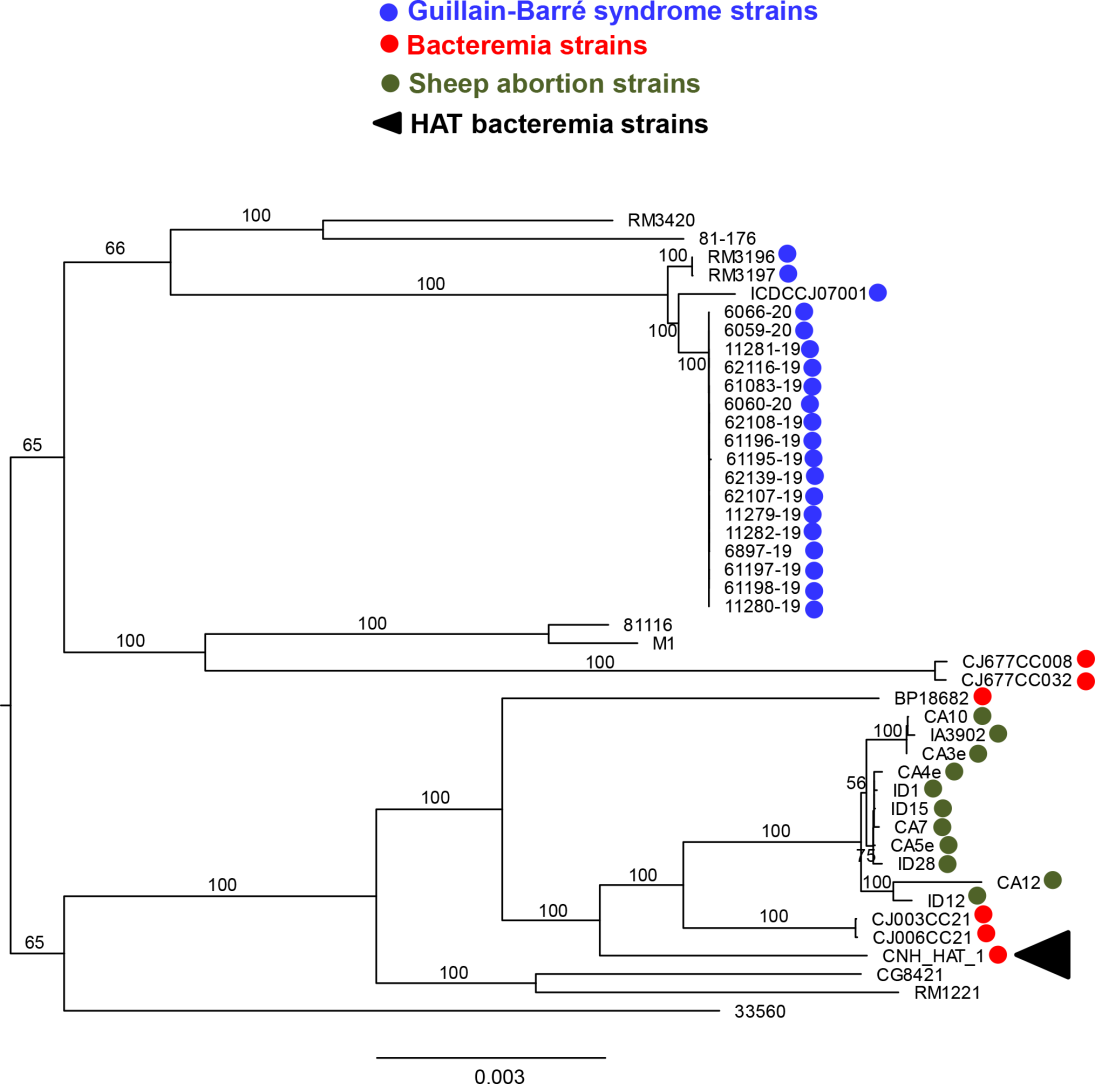
Phylogenetic analysis of whole genome sequence of CNH-HAT-1. CNH-HAT-1 (black arrowhead) was subjected to WGS using Illumina and Nanopore technologies, followed by phylogenetic analysis compared with different *C. jejuni* strains associated with different clinical complications. The Orthofinder (V2.5.5) was used for ortholog analysis of CNH-HAT-1 and representative *C. jejuni* genomes from NCBI to find single-copy genes. A maximum-likelihood-based phylogenetic tree with a bootstrap value of 1,000 iterations was built using Iq-tree 2.3.2. The scale bar indicates the number of amino acid substitutions per site. The tree was visualized using Figtree (v1.4.4). Blue dot, Guillian-barre syndrome strains; red dot, bacteremia-associated strains; green dot, sheep abortion strains.

Homology-based phylogenetic analysis using Orthofinder (V2.5.5) (18) revealed that CNH-HAT-1 is phylogenetically close to the recently reported invasive sheep abortion strains (for example, IA3902, green dots in Fig. 5), some bacteremia-associated *C. jejuni* isolates (mainly ST-21, red dots in Fig. 5), and RM1221, whereas it is relatively distant to ST-677 bacteremia-associated strains (CJ677CC008 and CJ677CC032, red dots in Fig. 5) and Guillain-Barré syndrome strains (blue dots in Fig. 5). Of note, the other blood ST-21 isolates, which are not HAT, are more closely related to the sheep abortion isolates than CNH-HAT-1 (Fig. 5). Therefore, CNH-HAT-1 is the first invasive blood *C. jejuni* isolate proven *in vitro* to be HAT.

## DISCUSSION

We identified and characterized phenotypically and genomically a HAT *C. jejuni* strain causing bacteremia from a B-ALL pediatric patient. Interestingly, *C. jejuni* can grow under aerobic conditions when cultivated statically, which may be due to the very limited diffusion of oxygen in a liquid medium (19), with the small amount of dissolved oxygen being rapidly depleted by bacterial growth (19–21). CNH-HAT-1 is resistant to penicillins and cephalosporins but susceptible to aminoglycosides, fluoroquinolones, and macrolides.

MLST analysis revealed that CNH-HAT-1 belongs to ST-21. However, ST-21 is diverse and not considered to be specific to any host or niche (22). In contrast, the predominant CC in Finnish bacteremia-associated strains is ST-677, which contains CJIE1 elements, although some strains have partially deleted CJIE1 sequences (15, 16). In an *in vitro* cell line infection model, CJIE1-positive strains were 6-fold to 7-fold more adherent and 16-fold to 21-fold more invasive compared with a CJIE1-negative strain (23). However, these findings are based on a limited number of strains, and the role of CJIE1 genes in bacteremia requires further investigation.

Our analysis showed that the gene for the enterobactin receptor CfrB was present in CNH-HAT-1. CfrB depends on a periplasmic enterobactin hydrolase Cee (24) for iron utilization, similar to the *Escherichia coli* enterobactin receptor IroN, which relies on the periplasmic enterobactin hydrolases IroD and IroE (25). Interestingly, *E. coli* IroN has been reported to promote the invasion of epithelial cells (26), suggesting that CfrB may also play a dual role in the iron uptake and in the invasion or translocation of the epithelial barrier. However, further investigation is needed to confirm this possibility.

This report represents the first description of a novel, invasive HAT *C. jejuni* strain causing bacteremia in humans. The phenotypic analysis characterized its HAT phenotype and AST profiles. The genomic analyses of CNH-HAT-1 shed light on some key genomic features linked with its pathogenesis. Despite its susceptible *in vitro* AST profile, the HAT *C. jejuni* isolate may pose more challenges to infection control. HAT isolates can survive in oxygen-rich environments longer, which increases the likelihood of transmission via food chain or in healthcare environments. Furthermore, HAT *C. jejuni* isolates are often more resistant to aerobic stress, therefore tolerating common disinfecting agents such as hydrogen peroxide or ozone. A clinical microbiology lab should be vigilant in detecting the emerging HAT *C. jejuni* stains causing bacteremia. Although culture-based organism identification remains the mainstay, more research is needed for rapid microbe identification, including bacteremia-causing HAT *C. jejuni* (27–33).

## Data Availability

An assembled genome (four contigs) was deposited to the NCBI BioSample database (http://www.ncbi.nlm.nih.gov/biosample/) under the accession number SAMN44101439. All other data are contained within the article. Data can be made available upon request of the lead contact.
